# Continuous intraoperative perfusion monitoring of free microvascular anastomosed fasciocutaneous flaps using remote photoplethysmography

**DOI:** 10.1038/s41598-023-28277-w

**Published:** 2023-01-27

**Authors:** Sebastian P. Schraven, Benjamin Kossack, Daniel Strüder, Maximillian Jung, Lotte Skopnik, Justus Gross, Anna Hilsmann, Peter Eisert, Robert Mlynski, Eric L. Wisotzky

**Affiliations:** 1 Department of Oto-Rhino-Laryngology, Head and Neck Surgery “Otto Körner”, Rostock University Medical Center, Doberaner Straße 137-139, 18057 Rostock, Germany; 2 Vision and Imaging Technologies, Fraunhofer Heinrich Hertz Institute HHI, Einsteinufer 37, 10587 Berlin, Germany; 3 Department of General, Visceral, Thoracic, Vascular and Transplantation Surgery, Rostock University Medical Center, Schillingallee 35, 18057 Rostock, Germany; 4 Visual Computing, Institut für Informatik, Humboldt-Universität zu Berlin, Unter den Linden 6, 10099 Berlin, Germany

**Keywords:** Translational research, Imaging and sensing, Surgical oncology

## Abstract

Flap loss through limited perfusion remains a major complication in reconstructive surgery. Continuous monitoring of perfusion will facilitate early detection of insufficient perfusion. Remote or imaging photoplethysmography (rPPG/iPPG) as a non-contact, non-ionizing, and non-invasive monitoring technique provides objective and reproducible information on physiological parameters. The aim of this study is to establish rPPG for intra- and postoperative monitoring of flap perfusion in patients undergoing reconstruction with free fasciocutaneous flaps (FFCF). We developed a monitoring algorithm for flap perfusion, which was evaluated in 15 patients. For 14 patients, ischemia of the FFCF in the forearm and successful reperfusion of the implanted FFCF was quantified based on the local signal. One FFCF showed no perfusion after reperfusion and devitalized in the course. Intraoperative monitoring of perfusion with rPPG provides objective and reproducible results. Therefore, rPPG is a promising technology for standard flap perfusion monitoring on low costs without the need for additional monitoring devices.

## Introduction

In reconstructive surgery, flap monitoring is crucial for early detection of perfusion problems. The survival of free flaps anastomosed to suitable donor vessels depends on adequate tissue perfusion. Flap failure may result from arterial or venous occlusion due to vasospasm, thrombosis, external compression, vessel kinking, or hematoma^1^. Mainly thrombotic events in the flow area of the microanastomosis lead to a total loss after a free microvascular tissue transfer, which occurs in 2–6% of cases^2,3^. Timely re-exploration can increase the rate of flap salvage. Today, detecting early signs of ischemia and consequently planning revision surgery is only possible with clinical postoperative monitoring^4,5^. Signs of ischemia can be caused by primary no flow, which is caused due to four possibilities: anastomosis insufficiency, arterial or venous thromboses and injury of the perforating vessels during flap preparation. While thromboses can be treated with heparin rinsing, the other two cases need surgical correction. As early tissue reperfusion determines flap survival, the flap assessment in the operation room may be the future alternative.

A variety of objective and subjective monitoring techniques have been reported. The implantable Cook-Swartz Doppler probe, an invasive method that directly measures the blood flow in the pedicle consisting of a silicone cuff containing a 20-MHz piezoelectric crystal^6^. In addition to complications associated with probe insertion (clot around the probe interrupting signal) and removal (retained wire, pedicle laceration during extraction), the Cook-Swartz implantable Doppler probe has a high rate of false-positives (12%) and poor negative predictive value (33.3%)^7^. In fluorometry, a fluorescent marker such as indocyanine green is injected intravenously to assess tissue perfusion in the near-infrared wavelength range. However, the marker is rapidly volatile and only short but no continuous perfusion assessment is possible^8–11^. Techniques like laser Doppler flowmetry^12,13^, microdialysis^14^, oximetry^15^, CO_2_ monitoring^16^, measurement of temperature^17^, glucose and lactate^18^, nuclear medicine^19^, pH measurement^20^, hydrogen clearance^21^ and tomographic approaches^12,13^ are similarly characterized by invasiveness and stationary or complex handling, with the need of either additional expensive hardware or techniques. Promising non-invasive, non-contact and non-ionizing methods as hyper/multispectral^22–26^, laser speckle^27,28^ and thermal imaging^29–31^ require additional (cost-intensive) equipment and hyperspectral imaging has limited intraoperative capability due to insufficient real-time ability^32^.

Due to these disadvantages, the mentioned promising techniques widely failed to establish in clinical routine. Therefore, current flap perfusion monitoring remains a subjective postoperative bedside assessment^1,33–35^. However, an expert-investigator may not be present at all times. For this reason, there is a need for an objective, reliable and examiner-independent method to assess flap perfusion^13^.

**Figure 1:**
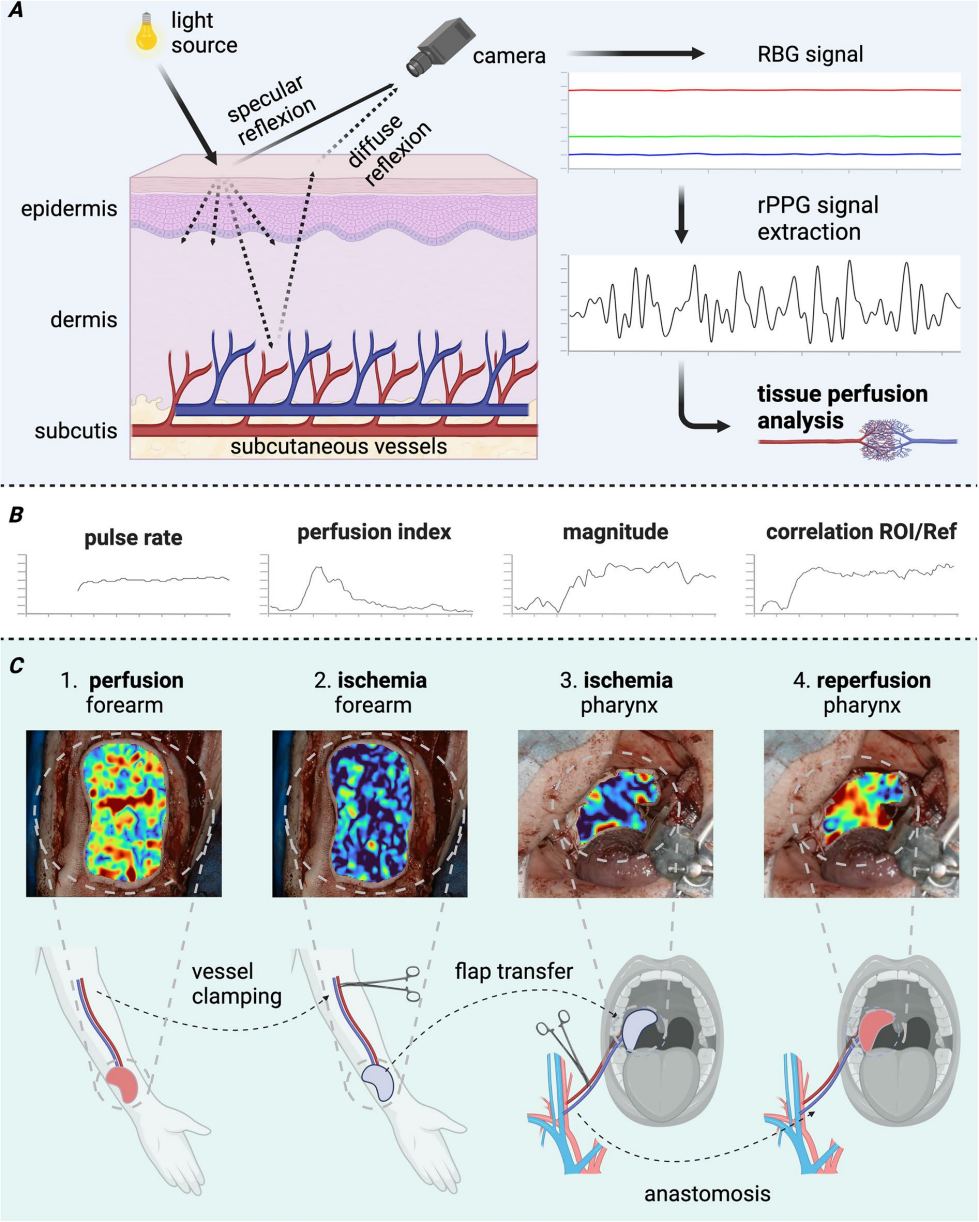
Intraoperative flap monitoring. (**A**) Method of remote photoplethysmography (rPPG) using the continuously captured RGB signal to extract the rPPG signal and analyze tissue perfusion. (**B**) The proposed parameter for flap perfusion monitoring. (**C**) The study setup with 1. monitoring of healthy well-perfused forearm area, 2. monitoring of prepared flap at forearm at the beginning of ischemia, 3. monitoring transferred flap at pharynx still non-perfused until 4. reperfusion. The visualization of the determined perfusion parameter allows a continuous intraoperative flap monitoring, here shown for #9 using parameter magnitude for all four analyzed time points.

Photoplethysmography (PPG), as a diagnostic method that relies on detecting the reflection of emitted red-infrared light, can directly measure red blood cell flow^36^. The principle is based on blood circulation and the fact that blood absorbs more light than surrounding tissue. Variations in blood volume affect light transmission and reflectance. Thus, PPG can be used as an objective and continuous surveillance method. Remote photoplethysmography (rPPG) or imaging photoplethysmography (iPPG) is a non-contact monitoring technique (Fig. 1A) providing the same information as PPG on physiological parameters in various medical applications such as (post-)operative tissue perfusion measurements and wound assessment^37–41^. Though the penetration depth of light into skin is low, e.g., approx. 2.5 mm for light at 810 nm and less for lower wavelength^42^, the behavior of the PPG signal is as well related to changes in cutaneous blood volume^43–45^. The method is based on the local signal characteristics and variations in classical red–green–blue (RGB) video recordings^46,47^ from which vital signs can be extracted^37^. In addition, local rPPG signals can be separated spatially. With this locally resolved information, specific spatial blood flow propagation can be analyzed from the signal-to-noise ratio (SNR) and pulse transit time (PTT)^48,49^. A good visualization of blood flow propagation and representation of the signal quality of each spatial position would allow the surgeon to make a quantitative diagnosis^50^.

The aim of this study is to analyze whether the use of rPPG is feasible for objective and reproducible monitoring of flap perfusion in patients after reconstruction with free fasciocutaneous flaps (FFCF) and to define thresholds for rPPG flap assessment.

## Results

We determined pulse rate and three additional parameters, (1) perfusion index (PI), (2) rPPG correlation $\rho$ (between rPPG signal of ROI and defined reference region), and (3) magnitude $M$ of the frequency component corresponding to the pulse rate, from the extracted rPPG signal. These three parameters allowed a significant differentiation between successfully transplanted and critically perfused flaps. For all 15 patients (11 male, 4 female; 63 ± 11 years of age), it was possible to quantitatively evaluate the process of reperfusion of the implanted FFCF based on the local rPPG signal and the three parameters. To observe and quantify signal quality, the SNR of the corresponding rPPG signal is analyzed. The ischemia time of the FFCF was 111 ± 31 min, cf. Table S1. All flaps except one were successfully transplanted and are healthy. Seven of these presented a successful flap transplantation without any specific incident. While seven were successfully implanted but showed shorter to longer (several seconds to approx. 1 min) deviations from the normal reperfusion during the surveillance. One flap (#8) showed no vitality during postoperative clinical inspection; to promote granulation formation from depth, the necrotic flap tissue was removed five weeks after surgery.

### Visualization of local fasciocutaneous flaps behavior

Analyzing the three parameters (PI, rPPG correlation, and magnitude) locally, i.e., at each pixel position within the region-of-interests (ROI), and mapping these parameters to the corresponding position within the video recording makes it possible to visualize the flap behavior over time. The visualization is presented exemplary for three different patients (#7, #9 and #13). In Fig. 1C top row, the magnitude $M$ is visualized at four specific time points: before and after clamping arteria at the donor side and before and after reperfusion at the final location in the oral cavity.

**Figure 2:**
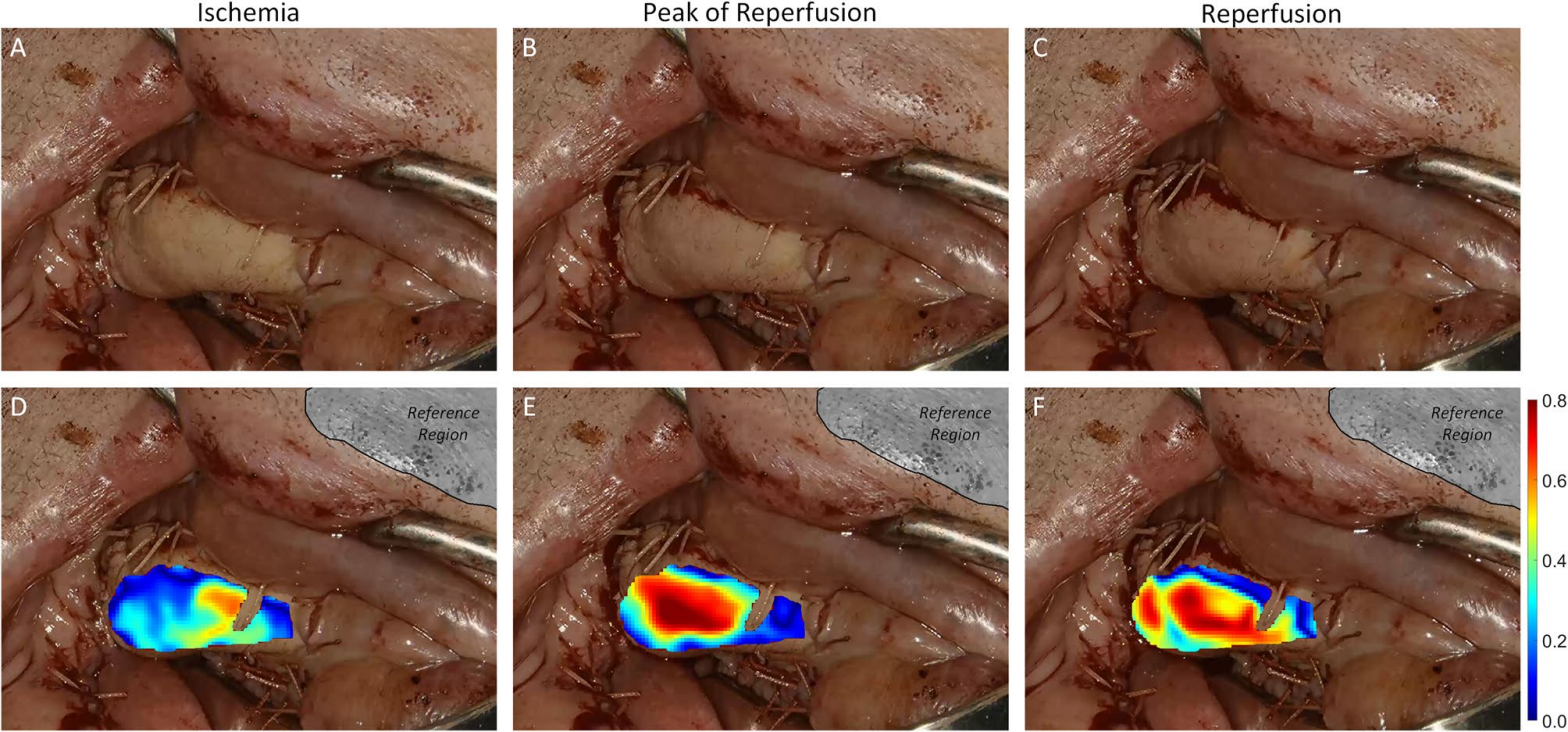
Visualization of local perfusion analysis. The third recording of #7 at three different time points (column-wise from left to right): Ischemia ($t=53$ s), Peak of Reperfusion ($t=78$ s), and during reperfusion ($t=137$ s). (**A**–**C**) show the standard RGB view. (**D**–**F**) show the same images augmented with local correlation analysis showing the local perfusion relationship between flap and reference region (gray). The local correlation strength is color-coded with the color bar on the right.

In Fig. 2, the visualization of $\rho$ (D–F) is shown in comparison to the standard RGB view (A–C) at three specific time points during reperfusion. The common RGB view of the surgeon (top row) is augmented with a ‘heat map’ showing the local rPPG correlation behavior over time. Thus, critical perfusion is locally and continuously detectable. In some small areas, within the ROI, short and small (frame-wise) abrupt changes of the heat map may occur. These changes can be caused by a strong noise component within the rPPG signal of the corresponding spatial position. However, long-time (over seconds) development of perfusion becomes visible for the surgeon intraoperatively.

It is possible to visualize all three analyzed parameters, e.g., the magnitude is selected for visualization in Fig. 1C. The visualization of the continuous perfusion behavior is shown in Movie S1 in the supplemental material. At the beginning, PI and rPPG correlation ρ are low (colored from blue to greenish). At the time of reperfusion, PI shows its characteristic peak (in red) and at the same time, the correlation ρ starts to increase (becoming red over the entire flap) showing continuous perfusion.

### Baseline perfusion analysis of flaps at donor side

**Figure 3:**
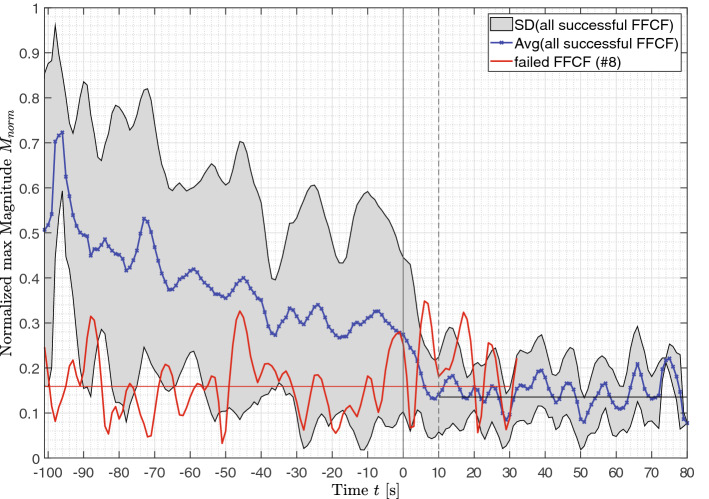
Extracted normalized magnitudes of all prepared flaps located at the forearm. The blue curve represents the mean of all flaps and the gray area is the SD ($n=14$). In comparison, the red curve shows the extracted normalized magnitude of failed case 8 (with its average as a horizontal red line). The data are normalized with the averaged magnitude of the analyzed healthy unprepared flaps (recording #1—flap baseline). The time point $t=0$ s marks the closure of the radial artery and the beginning of ischemia. After $t=10$ s, the used time window is completely in ischemia. The horizontal black line marks the ischemia baseline. The closure of the veins happened in the time window $t=-56.2s \pm 34.4$ s showing a first continuous decrease in magnitude values.

The perfusion analysis of the harvested flap at the donor side in the forearm is shown in Fig. 3 with the averaged normalized maximum magnitude ${\overline{M} }_{norm}({f}_{GT})$ of all successfully transplanted FFCFs as blue curve with a gray SD window. The magnitude $M$ is normalized using ${\overline{M} }_{baseline}=5472.2$ of healthy FFCFs, see section “Methods”, with frequency ${f}_{GT}$ represents the externally documented pulse rate. Following vein closure, ${\overline{M} }_{norm}{(f}_{GT})$ decreased continuously starting at perfused baseline ${\overline{M} }_{perf}({f}_{GT})=0.7063$ with 95% confidence interval (CI) of [0.4388, 0.9737] from approx. $t=-100$ s until closure of the artery at ${t}_{ischemia}=0$ s. After closure of the radial artery, the signal dropped for approx. $10$ s due to the chosen ${t}_{win}=10$ s and reached an ischemia baseline of ${\overline{M} }_{ischemia}({f}_{GT})=0.1538$ with 95% CI [0.1353, 0.1723]. Additionally, the failed flap is included in the Fig. 3 in red, showing a constant ${\overline{M} }_{failed}({f}_{GT})=0.171$ with 95% CI [0.159, 0.1911], which is comparable with the ischemia baseline, already before the closure of vein and artery, respectively.

In the following the three analyzed parameters are presented as average over all successfully transplanted FFCFs as well as representatively for two successfully implanted and incident-free FFCFs, i.e. #10 and #11, the failed #8 and two FFCFs showing shorter (for approx. 1 s, e.g., #6) to longer (up to 1 min, e.g., #14) incidents during the time of surveillance. The documented opening of the external carotid artery (ECA) distal to the anastomosis marks the reperfusion.

### Perfusion index (PI)

**Figure 4:**
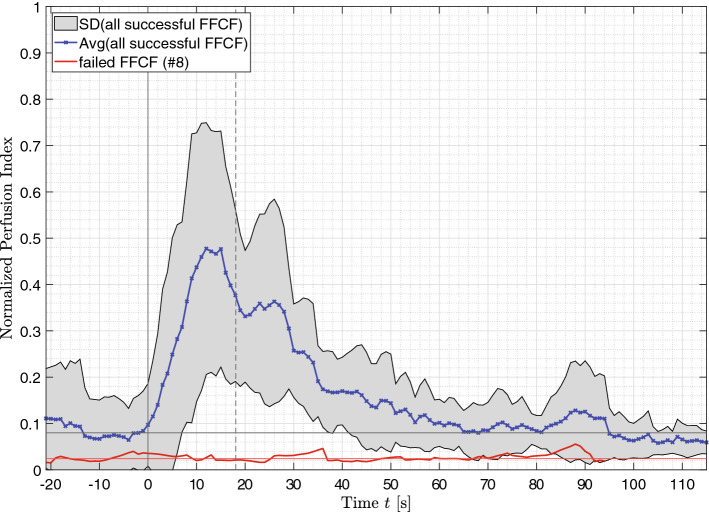
Perfusion index PI. The blue curve represents the average of all successfully transplanted FFCFs and the gray area includes the SD. In comparison, the red curve shows the PI of failed flap #8. The PI data are normalized with the highest extracted PI value in all data $P{I}_{max}=1.072$. The time point $t=0$ s marks the documented reperfusion. The reperfusion is detectable in the data after $t=18.08$ s (first detection of a pulse rate in the rPPG signal of the FFCF after reperfusion). The horizontal black line marks the baseline of PI of all successful reperfused flaps, while the horizontal red line marks the average of PI of the failed flap.

The reperfusion of the FFCF is detectable in the data at ${t}_{PI} =(18.083\pm 9.414)$ s after the documented reperfusion event. The detectable reperfusion of an FFCF correlated with a re-detectable rPPG signal of that FFCF. In Fig. 4, the average normalized PI of all successfully transplanted FFCFs is shown, where ${t}_{PI}=0$ s represents the documented time of reperfusion (ECA distal to the anastomosis was opened). The ECA was opened proximal to the anastomosis in average 9 s after opening distal.

**Figure 5:**
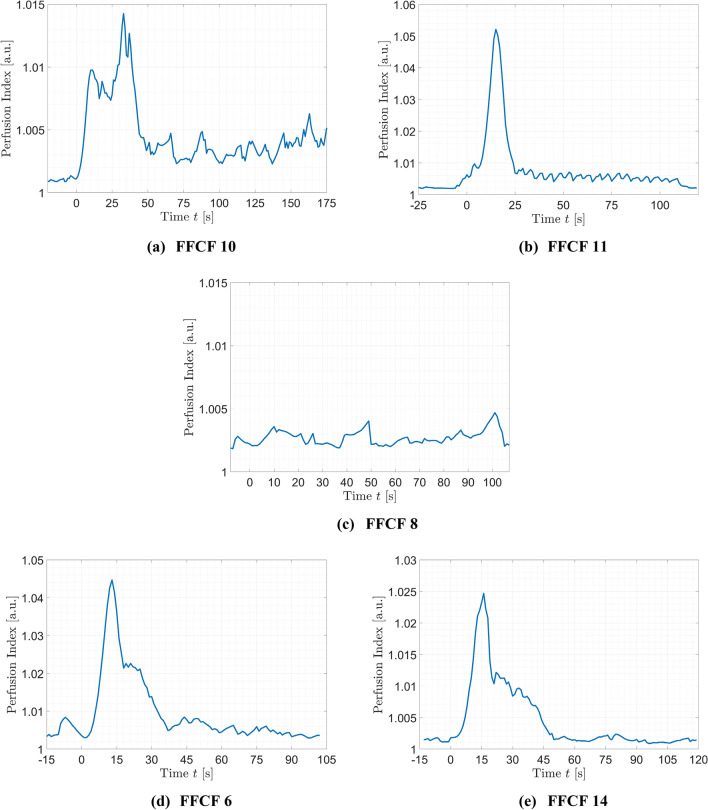
Perfusion index PI of selected cases. The documented reperfusion was set to $t=0$ s. (**a**) #10 and (**b**) #11 were successful, incident-free reperfusions showing a PI peak. (**c**) Failed flap (#8) showed no PI peak. (**d**) #6 and (**e**) #14 showed the expected PI peak although vasospasms occurred after reperfusion.

The PI was normalized with the maximum detected peak of all FFCFs $P{I}_{max}=1.072$. The peak of the averaged PI signal reached its maximum at ${t}_{PI}=15.33$ s with 95% CI [14.47, 16.2], which corresponds to the first detectable pulse rate in the rPPG signal of the FFCF after reperfusion. In the time window of 20–30 s, i.e., 5–15 s after the first peak, a second peak was detectable. This second peak in PI correlated with the opening of the ECA proximal to the anastomosis and is in average at ${t}_{PI}=25.72$ s with 95% CI [24.92, 26.52]. After $35$ s to $40$ s, the reperfusion process was completed showing a dropping of the perfusion intensity to a perfused baseline $P{I}_{perf}$. In contrast, the PI of failed flap showed no perfusion peak after reperfusion (cf. Fig. 5c), and the overall normalized signal with ${\overline{PI} }_{failed}=0.024$ with 95% CI [0.0216, 0.0267] was always below the averaged PI signal of successfully transplanted FFCFs ($P{I}_{perf}=0.080$ with 95% CI [0.068, 0.092]). Further, no robust pulse rate was detectable during the recording for the failed flap.

For #10 and #11, the opening of the ECA proximal to the anastomosis after first reperfusion was 22 s and 2 s, respectively. A first robust pulse rate was detectable at ${t}_{\#10}=22$ s and ${t}_{\#11}=13$ s, matching the ongoing measured ground truth pulse rate robustly afterward. The two peaks in PI of #10 (Fig. 5a) have a period of 22 s ($PI\left({t}_{1}=11 \mathrm{s}\right)=1.010$ and $PI({t}_{2}=33 \mathrm{s})=1.014$) and correlate with opening the individual arterial branches. For #11, the two incidences had a time interval of only 2 s, and as such, the two peaks cumulatively overlap (Fig. 5b), visible in the increased peak maximum with $PI(t=15 \mathrm{s})=1.052$. The non-perfused baseline of the PI was $P{I}_{ischemia}=1.001$ for #10 and $P{I}_{ischemia}=1.003$ for #11, respectively. The perfused baseline for PI as well as correlation ρ can be analyzed after the reperfusion is finished, i.e., starting with ${t}_{\#10}=47$ s and ${t}_{\#11}=26$ s. For PI, the perfusion baseline was $P{I}_{perf}=1.002$ for #10 and $P{I}_{perf}=1.008$ for #11. The PI of FFCFs showing shorter (for approx. 1 s, e.g., #6) to longer (up to 1 min, e.g., #14) incidents (Fig. 5d,e) was similar to the incident-free flap behavior.

### Correlation of rPPG

**Figure 6:**
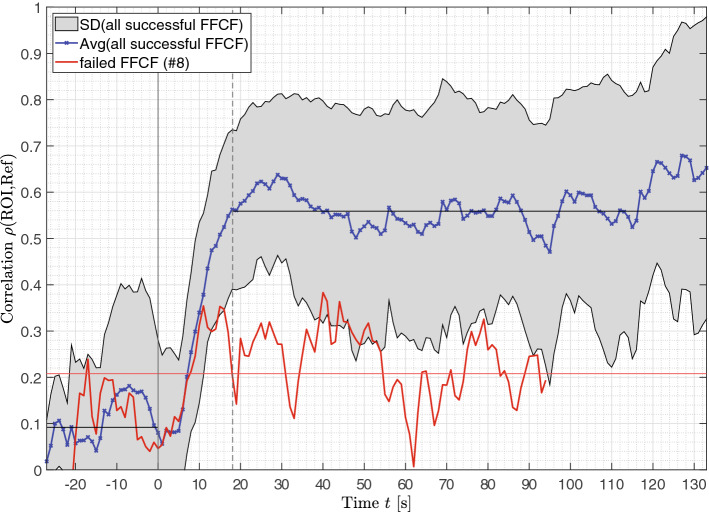
rPPG correlation ρ. The blue curve represents the average of all successful transplanted FFCFs and the gray area includes the SD. In comparison, the red curve shows the correlation of failed flap (#8). The time point $t=0s$ marks the documented reperfusion. The reperfusion is detectable in the data after $t=18.08$ s. The horizontal black line until t = 0 s marks the average correlation of ischemia with 0.09, while the average correlation after reperfusion is 0.56. The horizontal red line marks the average correlation of the failed flap with $\overline{\rho }=0.208\pm 0.090$.

**Figure 7:**
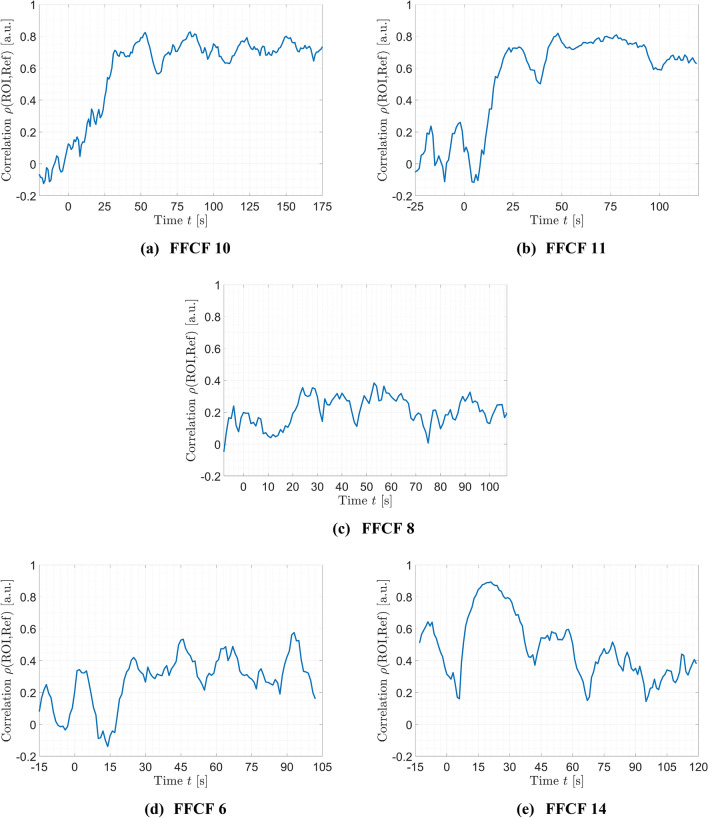
rPPG correlation ρ of selected cases. The documented reperfusion was set to $t=0$ s. (**a**) #10 and (**b**) #11 were successful, incident-free reperfusions showing an increasing correlation starting at reperfusion remaining continuously high afterwards. (**c**) The correlation of the failed flap (#8) remained at the ischemia level. (**d**) #6 and (**e**) #14 showed a correlation increase after reperfusion. Due to vasospasms, the correlation dropped in the further course to a lower level.

The averaged correlation $\rho$ between the ROI and a reference skin region is shown in Fig. 6. Again, the average was determined using only the successfully transplanted FFCFs. The failed flap is shown individually in red. It is visible that $\rho$ was increasing when reaching $P{I}_{max}$. At that time, the correlation increased from the non-perfused baseline ${\overline{\rho }}_{ischemia}=0.092\pm 0.060$ to the perfused baseline of ${\overline{\rho }}_{perf}=0.559 \pm 0.034$. The beginning of the perfused baseline ${\overline{\rho }}_{perf}$ correlates with the time ${t}_{PI}=18.08$ s when perfusion is detectable. A significant difference of $\Delta \overline{\rho }=0.467$ is obtained between ischemia and perfusion ($p<0.001$). Though, vasospasms were detectable in the time window up to $t=110$ s for several FFCFs, resulting in a decreased correlation for the time of the spasm and the specific FFCF. The failed flap (#8) showed an average correlation through the entire monitoring time of $\overline{\rho }=0.208 \pm 0.090$, with insignificant differences before and after reperfusion showing ${\overline{\rho }}_{ischemia}=0.115 \pm 0.063$ and ${\overline{\rho }}_{perf}=0.239\pm 0.075$, respectively (cf. Fig. 7c).

For the incident-free flaps (e.g., #10 and #11) in the time of reperfusion, the correlation reached ${\rho }_{perf}=0.707$ with 95% CI [0.677, 0.736] and ${\rho }_{perf}=0.728$ with 95% CI [0.684, 0.772], respectively, while ${\rho }_{ischemia}\approx 0$ for both cases (cf. Fig. 7a,b). The correlation $\rho$ of FFCFs showing specific incidents was increasing after reperfusion as described for the optimal cases. However, $\rho$ was decreasing after some time, as shown for #6 and #14 in Fig. 7d,e, respectively. This behavior corresponded with dropouts in pulse rate detection for #6 at $t=27s, 56-57$ s, and $67$ s as well as for #14 at $t=62-74$ s and $78-106$ s. The long time window of no rPPG signal in #14 resulted in a correlation drop to ${\rho }_{ischemia}$. At the end, the pulse rate was detectable again, indicating the end of the incident.

### Magnitude and signal-to-noise-ratio

**Figure 8:**
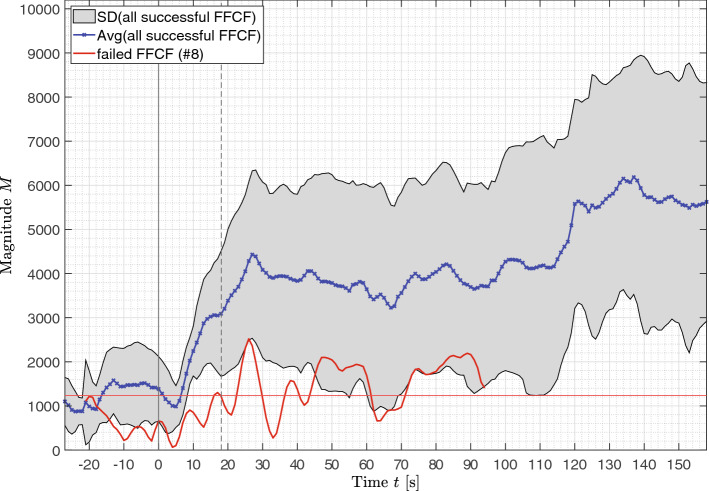
Magnitude M. The blue curve represents the average of all successful transplanted FFCFs and the gray area includes the SD. In comparison, the red curve shows the correlation of failed flap (#8). The time point t = 0 s marks the documented reperfusion. The reperfusion is detectable in the data after $t=18.08$ s. The horizontal red line marks the average magnitude of the failed flap with $\overline{M }=1234.1\pm 639.7$, which corresponds to the average magnitude of the ischemic FFCF ${\overline{M} }_{ischemia}=1246.6\pm 224.7$.

The average magnitude $M$ is shown in Fig. 8. As before, the average was determined using only the successfully transplanted FFCFs. The failed flap is shown individually in red. It is visible that $M$ was increasing when reaching $P{I}_{max}$. At that time, the magnitude increased from the non-perfused baseline ${\overline{M} }_{ischemia}=1246.6\pm 224.7$ to a first perfused baseline plateau of ${\overline{M} }_{perf}=4065.9\pm 524.6$. The beginning of the first perfused plateau ${\overline{M} }_{perf}$ correlates with the time ${t}_{PI}=18.08$ s when perfusion is detectable. A significant difference of $\Delta \overline{M }=2819.3$ is obtained between ischemia and perfusion ($p<0.001$). As vasospasms were detectable in the time window up to $t=110$ s for several FFCFs, the average magnitude further increased at $t>110$ s. The failed flap (#8) showed with $\overline{M }=1234.1\pm 639.7$ the same average magnitude as ${\overline{M} }_{ischemia}$.

**Figure 9:**
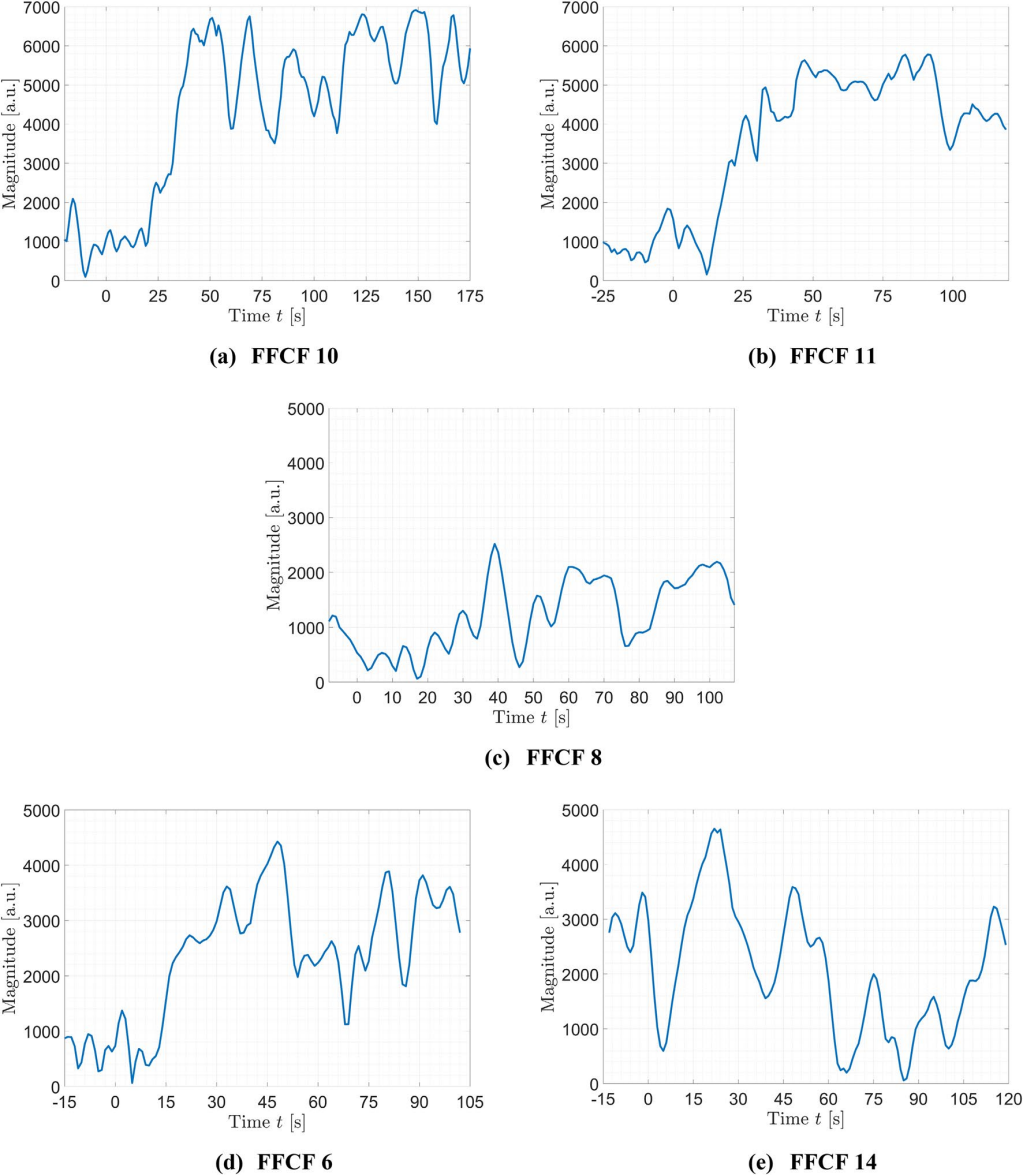
Magnitude of selected cases. The documented reperfusion was set to $t=0$ s. (**a**) #10 and (**b**) #11 were successful, incident-free reperfusions showing an increasing magnitude starting at reperfusion remaining continuously at the perfusion baseline. (**c**) The magnitude of the failed flap (#8) remained at the ischemia level. (**d**) #6 and (**e**) #14 showed an inconclusive magnitude behavior after reperfusion due to vasospasms.

Magnitude (Fig. 9) and SNR showed a similar trend and are comparable to the trend of correlation $\rho$. The curves were increasing at the time of the first robust detectable pulse frequency. The magnitude then reached a baseline ${M}_{perf}=5562.4$ for #10 and ${M}_{perf}=4788.9$ for #11, which is in the range ${\overline{M} }_{baseline}=5472.2$ of the healthy FFCF region before harvesting from the donor site. Likewise, the magnitude dropped if incidents, e.g. vasospasm, occurred. For the failed flap, the magnitude remained in the range of the non-perfused baseline. The signal quality in terms of SNR fluctuated around $-6$ to $0$ dB after reperfusion and between $-14$ and $-8$ dB before reperfusion.

For the flaps showing occurring incidents, it would be of interest to show how the transplanted flap behaves after one hour of reperfusion. The same interest applies for the failed case, which showed no sign of perfusion in the entire recording *flap reperfusion*. After one hour of reperfusion, the magnitude of #14 as example of incident afflicted flap showed a well-perfused behavior with a high signal quality $\overline{SNR }=0.263$ dB, while the failed flap showed a limited magnitude with average signal quality $\overline{SNR }=-3.512$ dB, although in both cases, it was possible to detect the pulse rate in the FFCF.

## Discussion

The present study shows the ability of rPPG to continuously monitor and evaluate the perfusion of transplanted FFCFs. We performed measurements using a standard operating microscope. While, the data analysis was executed postoperatively, rPPG as a non-contact vital sign measurement technique was used for the first time for intra- and postoperative assessment of perfusion in FFCFs. Sufficient reperfusion in FFCFs resulting from the opening of the external carotid artery was clearly detectable. Additionally, it was possible to distinguish intermittent restrictions of perfusion such as vasospasm and insufficient perfusion leading to flap failure.

We recommend three parameters to evaluate the perfusion quality robustly: (1) perfusion index (PI), (2) correlation $\rho$ of the analyzed rPPG signal with a reference rPPG signal, and (3) magnitude of the FFCF. The detected pulse rate is clinically no robust parameter as for long-term surveillance it is possible to detect a pulse rate even in not suitable perfused (non-functional) flaps, cf. the failed flap in Fig. S4c. This is possible as also in non-functional flaps blood flow occurs from the arteria through the vein, but without supplying the flap tissue^51^.

The surgeon can use PI evaluation to detect the time of reperfusion that visualizes the effect of hyperemia dissolving single artery openings. Therefore, PI is a reliable indicator if the expected reperfusion is occurring. The peak in PI after the opening of the anastomosis with the subsequent drop likely represents an ischemia–reperfusion phenomenon. This is explained by reactive hyperemia and involves temporary widening of the arterioles to compensate for the tissue hypoxia during ischemia. Following this peak, we observed an expected normalization of the perfusion index. In contrast, magnitude allows robust long-term surveillance, as indicated by the analysis one hour after reperfusion, cf. Fig. 9 and Fig. S4. The SNR allows data quality monitoring and was consistently above other reported rPPG SNR ($SNR \sim 0$ dB for perfused flaps and $SNR\sim -10$ dB for ischemic flaps vs. $SNR$ between $-6$ and $-17$ dB for perfused faces)^52^.

The parameter correlation $\rho$ requires the presence of reference information, either a recorded skin region or the externally measured pulse rate. If a reference region is chosen in the field of view, the selection of the reference must consider perfusion restrictions through intraoperative interruption of blood supply. Using external pulse rate can lead to less accurate results, since the PPG signal has to be approximated using a sine wave, which is only a rough estimate of the true PPG waveform. Likewise, this approach is susceptible with regard to the sine wave parameters, especially the phase angle; an incorrect value leads to inaccurate results. However, both cases give evidence of perfusion differences in the FFCF.

In short time intervals after reperfusion, deviation from the optimal course could be observed in the data. These deviations were due to vasospasms. Therefore, the surveillance time (recording length of video 3 *flap reperfusion*) was increased after the first three patients to a minimum of 150 s after reperfusion. The vasospasms highlight the advantage of continuous monitoring. The incidence of vasospasm in microsurgical anastomoses, leading to a reduction in blood flow and an increased tendency to thrombus formation, is reported to be 5–10%^53^. The pathogenesis of vasospasms is not fully understood. Factors such as mechanical stretching of the vessel wall, endothelial damage, low ambient temperature, pH, anatomic location, and hematoma formation are thought to contribute to this phenomenon^54^. Vascular spasms may occur intraoperatively shortly after the release of the vascular clamp, following microvascular anastomosis but also within 48–72 h postoperatively^55^. Early recognition of intraoperative vasospasm is important because it can be treated easily using vasodilators^56^. The isoquinoline alkaloid papaverine and the calcium channel blocker nicardipine exert their effects locally and distally in the microcirculation of the flap^57^.

Thus, intraoperative deviations from the expected PI, ρ, $M$ may be an indication for revision of the anastomosis as an immediate surgical consequence and would allow the detection of possible flap failure. This study does not present any thresholds for the three parameter PI, ρ, $M$ to robustly differentiate between perfused and ischemic FFCFs. Further studies with a larger patient cohort (especially with a larger number of failed flaps) are needed to determine robust limits. Also a method to condensate the three parameter to one ischemic status value would simplify the visualization task.

The data of the failed transplanted flap indicate that flap failure may be detectable already at the donor site. In FFCFs, the skin area is supplied by small perforator vessels extending from the main vascular pedicle. Most likely, in the failed flap, these perforating vessels were harmed during flap preparation. This shows, that intraoperative monitoring of flap perfusion is already necessary during flap harvesting. If perfusion is impaired when the pedicle flap is lifted, the perforator vessels may be damaged and the flap is not suitable for transplantation. After insertion of the FFCL into the defect to be reconstructed, it is recommended to start with the anastomosis of the artery. If blood emerges from the venous vascular pedicle, problems with the arterial anastomosis as well as thromboembolic events of the arterial and venous vessel can be excluded. Continuous monitoring during venous anastomosis can detect insufficiency by a change in flap perfusion. Thus, intraoperative monitoring has the potential of immediate surgical corrections that lacks in mere postoperative monitoring. However, to make more accurate conclusions about the exact behavior of failing flaps, a study with more patients would be needed. Only one failing free flap in this study corresponds to the documented transplanted flap failure of 2–6%^2,3^.

The visualization of the proposed parameter enables the surgeon to analyze the quality of the FFCF at the sampling site as well as to detect critical perfusion after reperfusion. An integration of this visualization into existing image-guided systems could be a worthy assistance for the surgeons^58–60^. The apparent specific perfusion incidents during continuous monitoring (e.g., short occurring vasospasms) can be efficiently addressed by using vasodilators. Even in the case of critically perfused FFCF, the procedure provides the surgeon with intraoperative indications for revision of the anastomosis in order to save the FFCF and the surgical success. Due to the local visualization of the flap perfusion, it would become possible to correct specific partial perfusion problems (e.g., caused by flap kinking) to enhance transplantation outcome.

Optimization of the method would allow real-time intraoperative detection of complications for immediate anastomotic revision. The value of the method compared with competing procedures is its widespread availability: as a camera-based procedure, rPPG can be performed with standard equipment in any operating room (e.g., with the surgical microscope). Automation of data analysis and comparative clinical studies with alternative technologies, like spectral analysis^61^, are needed before the widespread use of the procedure. Further studies are needed to establish the procedure for postoperative monitoring of flap perfusion.

In summary, this study could reveal flap failures at an early stage without the need for further complex and invasive methods. We could define the breakdown between flaps showing perfect reperfusion, specific incidents (e.g., vasospasm) during reperfusion and complete failure. This is going beyond results that have been recently published using a hyperspectral camera postoperatively for free flap monitoring^24,62,63^ as these only allow perfusion analysis at discrete time points postoperatively. In contrast, our image-based setup allows continuous intraoperative perfusion analysis that is essential to robustly record perfusion deviations at an early stage. An existing digital imaging unit, such as a surgical microscope or endoscope^58^, could be expanded for real-time intraoperative flap monitoring, while for postoperative monitoring commercially available cameras (e.g., in smartphones) could be used.

## Methods

### Study design

Between 05/2021 and 03/2022, patients, that underwent head and neck squamous cell carcinoma resection and defect reconstruction with an FFCF at a tertiary hospital in a Head and Neck Cancer Center setting, were included. Informed oral and written consent had been obtained appropriate to the decision of the institutional review board of the local ethical committee of the Rostock University Medical Center and all experimental protocols were approved by the institutional review board of the local ethical committee of the Rostock University Medical Center (A 2021-0187). The study was performed in accordance with the relevant guidelines, regulations and in accordance with the Declaration of Helsinki. It was split into four steps, see Fig. 1B.

Imaging and examinations took place within the regular routine of a tumor surgery. Fifteen patients were included in the study (Table S1). A high-resolution, fully digital surgical microscope (ARRIscope Evo2 ENT, MSI, Munich, Germany) was used for imaging during surgery using a frame rate of $60$ fps. The video resolution was $1920\times 1080$ pixels. The LED light source is a compound cluster of four different LEDs ranging from 440 to 640 nm^63^.

After tumor resection and neck dissection, an FFCF was elevated from the forearm for defect reconstruction. Prior to preparation, the flap was outlined by the surgeon and a baseline recording was taken with the microscope for 60 s at the donor site (*flap baseline*). Following exposition of the radial artery and the accompanying veins of the FFCF, the blood supply to the flap (ischemia) was recorded with the microscope (*flap ischemia*) for at least 30 s. Then, the collateral veins were clamped first, followed by the radial artery after approx. 30 s. For one patient, the radial artery was clamped first, followed by venous clamping after 22 s. Clamping of the radial artery at the forearm and the associated ischemia were recorded for at least 30 s. The flap vessels were rinsed with heparin saline solution in order to avoid microthromboses during ischemia time.

After reconstruction of the defect with the FFCF, microvascular anastomoses of the radial artery to the ECA (end-to-side) and the accompanying veins to the internal jugular vein (end-to-side) were performed. The opening of the clamped ECA and the associated reperfusion of the implanted FFCF via the anastomosed radial artery was again documented with the microscope (*flap reperfusion*). Here, care was taken to document ischemia of the implanted flap for at least 30 s before reperfusion. Then, the ECA was opened distally to the anastomosis and 5–10 s later the ECA was opened proximally. The final reperfusion of the flap was documented for at least 2 min to evaluate the direct evolution of flap reperfusion.

Consequently, three videos were recorded with the microscope for each patient. All videos were recorded at 60 fps. For validation reasons, the patient vital signs were recorded continuously and synchronized with each video recording.

### Data and setup

Postoperatively, we analyzed all three recorded surgical videos individually. In all recordings, the initial frame for evaluation was first selected and annotated by the surgeon. In *flap baseline*, the annotation of the ROI included the intraoperatively marked skin region. In *flap ischemia*, the annotated ROI was the prepared flap on a compress. In *flap reperfusion*, two ROIs were annotated, the implanted flap and a skin region within the face of the patient contralateral to the host site as a reference.

The video frames were motion-compensated using rigid body registration as breathing, heartbeat, and external physical contact caused slight movements in the video recordings. As the next step, for each registered ROI (flap or reference) the rPPG signal was extracted and the pulse rate was determined as described previously in Kossack et al.^65^. In this work, we distinguished between three different pulse frequencies: (a) ${f}_{GT}$ as ground truth, measured with an external patient monitor (pulse oximeter connected to the patient), (b) ${f}_{FFCF}$, extracted from the rPPG signal of the FFCF (i.e. ROI), and (c) ${f}_{Ref}$, extracted from the reference skin region, where ${f}_{GT}\approx {f}_{Ref}$ showing a difference only due to the different monitoring techniques, see Fig. S4c,f.

Using this extracted rPPG signal and pulse frequency, three indices were determined to analyze continuous flap perfusion: (1) rPPG correlation $\rho$ between flap and reference, (2) the magnitude $M$ of the rPPG signal in the frequency domain at ${f}_{GT}$ (reference rPPG signal), and (3) perfusion index (PI). The SNR was calculated to determine the signal quality.

The rPPG signal was extracted for all ROI time series locally using POS transformation^66^, as it was shown that POS transformation provides the most robust results under different lighting conditions^67^. For each image, the color values of each ROI were averaged for the individual color channels and concatenated with the averaged signal values of the previous images. Thus, for each color channel, a three-dimensional normalized time signal $\left(r(t),g(t), b(t)\right)$ was obtained. The rPPG signal $v$ was then determined using

1 $$v={X}_{s}+\frac{\sigma ({X}_{s})}{\sigma ({Y}_{s})}{Y}_{s}$$

with

2 $${X}_{s}=g(t)-b(t)$$

and

3 $${Y}_{s}=-2 r\left(t\right)+g(t)+b(t),$$

where $\sigma$ is the standard deviation (SD). To extract the pulse frequency from these waveforms, a fast Fourier transform (FFT) is applied and the magnitude is calculated. The frequency component with the highest magnitude represents the global pulse frequency. A sliding window ${t}_{win}$ was used to determine the pulse rate^49,68^. After every calculation, the sliding window was moved forward by ${t}_{step}$, and the calculation was repeated. Technically, it had been found that a sliding window size of ${t}_{win}=10$ s provided the most robust results. Therefore, all results are based on the analysis with a sliding window of ${t}_{win}=10$ s and step size ${t}_{step}=1$ s. The determined pulse rate was validated against the externally synchronously monitored vital signs.

The rPPG correlation $\rho$ was calculated requiring the rPPG signal of a reference skin region and the FFCF. The analyzed ROI and reference facial skin regions of the third recordings are presented in Fig. S1. As reference region a clean and directly camera facing skin surface is chosen. It is important that this region and their blood vessels is not influenced by the surgical procedure. If no reference region could be specified, it would be possible to use the external pulse rate measurements from patient monitoring to calculate a reference PPG signal using a sine wave^48,49^. For #3, it was not possible to define a reference region. In #1, the definition of a continuously perfused reference region failed. In both cases, the correlation was determined using a sine wave with the frequency ${f}_{GT}$ of the externally documented pulse rate as reference.

Theoretically, it is always possible to use this specific reference calculation. However, a reference skin region in a recording was used in this study if possible, as it provides a clear and robust rPPG signal for analysis and is computationally less expensive. The extracted rPPG signal of the reference region was correlated with the rPPG signal of the flap ROI by

4 $$\rho \left({v}_{j},{v}_{k}\right)=\frac{1}{N-1}\sum_{i=1}^{N}\left(\frac{{v}_{j,i}-\mu ({v}_{j}) }{\sigma ({v}_{j})}\right)\left(\frac{{v}_{k,i}-\mu ({v}_{k}) }{\sigma ({v}_{k})}\right),$$

where ${v}_{j}$ and ${v}_{k}$ represent the vital sign signals (rPPG signal) of two different ROIs, $i$ is the sample index in the time domain, $\mu$ is the mean, and $\sigma$ is the SD.

Further, the SNR of the local rPPG signal was calculated using^69^

5 $$SNR=10\,{\mathrm{log}}_{10}\left(\frac{{\sum }_{k={f}_{1}}^{{f}_{2}}{\left({U}_{m}\left(k\right) M\left(k\right)\right)}^{2}}{{\sum }_{k={f}_{1}}^{{f}_{2}}{\left(\left(1-{U}_{m}(k)\right) M\left(k\right)\right)}^{2}}\right)$$

to quantify the signal quality, with

6 $${U}_{m}(k)=\left\{ \begin{array}{c}1,\,\,\,for \left|f\pm 0.05Hz\right| \\1, \,\,\,for \left|2f\pm 0.05Hz\right| \\ 0,\,\,\,\,otherwise \end{array}.\right.$$

The rPPG signal of the ROI was transformed into the frequency domain via an FFT receiving the pulse frequency $f$. Either the pulse frequency ${f}_{ref}$, determined from the reference region, or ${f}_{GT}$, measured externally, can be used for $f$. In order to account for the heart rate variability (HRV) of the patient^43,70^ during ${t}_{win}$, a margin of ± 3 beats per minute (BPM) (or ± 0.05 Hz in the frequency domain) was added to the fundamental pulse frequency $f$ in Eq. (6) (pointed out by the global pulse frequency) and its second harmonic. This obtained spectral interval was defined as signal, i.e., ${U}_{m}=1$. The remaining frequency components were classified as noise $(1-{U}_{m})$, cf. Eq. (5). Additionally, the magnitude $M$ of the signal component $f$ was selected for further processing. In this study, $M({f}_{GT})$ was used for analysis.

The PI was calculated as the ratio between the mean and maximum amplitude of the rPPG signal in the specific time window according to Rasmussen et al.^71^ but using the normalized green value ${G}_{n}$^48,68^

7 $${G}_{n}=\frac{1}{N}\sum_{i=1}^{N}\frac{{g}_{i}}{{r}_{i}+{g}_{i}+{b}_{i}},$$

where ${r}_{i}$, ${g}_{i}$, and ${b}_{i}$ are the pixel-wise RGB components of the image frame. The value ${G}_{n}$ (Eq. 7) was low-pass filtered afterward.

To visualize the analyzed information intraoperatively, each parameter can be locally resolved augmented onto the frame using pseudo color coding from blue (low perfusion) to red (high perfusion). Then, the estimated signal was assigned to each frame within the one second time shift with interpolation.

Based on the first recording *flap baseline* of the healthy FFCFs, the magnitude at ${f}_{GT}$ of the well-perfused skin transplant tissue was calculated as baseline. Over all patients and the entire recording time, an average magnitude of ${\overline{M} }_{baseline}({f}_{GT})=5472.2$ was determined. This baseline was used to normalize the detected $M{(f}_{GT})$ in the second recording *flap ischemia*. All extracted $M$ were aligned at time ${t}_{ischemia}=0$ s, when the radial artery was clamped. Beforehand, the veins were clamped successively in a time window of ${t}_{vein}=-\left(56.2\pm 34.4\right)$ s. After insertion of the FFCF into the defect to be reconstructed and microvascular arterial anastomosis, reperfusion was applied by opening the ECA distal to the anastomosis and then proximal. The time of reperfusion was documented as $t=0$ s.

### Statistical analyses

We collected all data using an electronic database system (Microsoft Excel, Microsoft Corp., WA, USA). Statistical analyses were performed for each patient between different time points on specific defined anatomical regions using MATLAB R2020b (MathWorks, Inc., MA, USA). Descriptive statistics were applied for the complete dataset. For continuous variables and the three introduced criteria, the mean, SD, minimum, median, and maximum were calculated. In addition, the absolute and relative incidence of categorical variables is presented. Normalization with detected baselines had been applied. Polynomial and Gaussian fitting were used to determine specific trends. Changes over time were assessed using Friedman tests, while specific cases were compared using the Mann–Whitney U test. Results of statistical tests must be considered as descriptive.

## Supplementary Information

Supplementary Video 1.

Supplementary Information 1.

## Data Availability

The data that support the findings of this study are available on request from the corresponding author (SPS, ELW). The data is not publicly available due to state restrictions as it contain information that could compromise participant privacy and require consent.

## Abbreviations

ACC: Adenoid cystic carcinoma
BPM: Beats per minute
CI: Confidence interval
ECA: External carotid artery
EO: Enoral
FFCF: Free fasciocutaneous flap
FFT: Fast Fourier transform
iPPG: Imaging photoplethysmography
LP: Lateral pharyngotomy
MFR: Midfacial resection
MS: Mandibular splitting
PI: Perfusion index
PPG: Photoplethysmography
PTT: Pulse transit time
RGB: Red–green–blue
ROI: Region-of-interest
rPPG: Remote photoplethysmography
SCC: Squamous cell carcinoma
SD: Standard deviation
SNR: Signal-to-noise ratio
