# Supplementary material for: Continuous intraoperative perfusion monitoring of free microvascular anastomosed fasciocutaneous flaps using remote photoplethysmography
Source: Sci Rep. 2023 Jan 27;13:1532. doi: 10.1038/s41598-023-28277-w (PMC9883527; doi:10.1038/s41598-023-28277-w)
Supplement: Supplementary file 2 — Supplementary Information 1. [file 41598_2023_28277_MOESM2_ESM.pdf]

Supplemental Material

no.	sex	age (years)	localization	TNM stage	grading	UICC stage	histology	p16-status	tumor resection	C2 abusus	nicotine abuse (PY)	flap ischemia (min.)
1	♂	51	oropharynx	rpT3 pN0 L0 V0 Pn0 R0	G2	II	SCC	pos.	MS	no	15	94
2	♂	57	oropharynx	pT2 pN0 L0 V0 Pn0 R0	G3	II	SCC	neg.	EO	yes	30	115
3	♂	74	oropharynx	pT2 pN1 L0 V0 Pn0 R0	G3	I	SCC	pos.	EO, LP	yes	30	175
4	♀	75	oropharynx	pT2 pN1 L0 V0 Pn0 R0	G3	I	SCC	pos.	EO	no	0	80
5	♀	53	oropharynx	pT1 pN1 L1 V0 Pn0 R0	G3	I	SCC	pos.	EO, LP	yes	35	115
6	♂	69	oropharynx	pT1 pN0 L0 V0 Pn0 R0	G2	I	SCC	neg.	EO	yes	20	77
7	♂	68	oropharynx	pT1 pN0 L0 V0 Pn0 R0	G2	I	SCC	neg.	EO, LP	yes	30	92
8	♀	70	oropharynx	rpT1 pN0 L0 V0 Pn0 R0	G3	I	SCC	neg.	EO	yes	20	105
9	♂	78	oropharynx	pT2 pN3b L1 V1 Pn0 R1	G3	IVB	SCC	neg.	EO	yes	50	106
10	♀	55	oropharynx	pT2 pN1 L0 V0 Pn0 R0	G2	I	SCC	pos.	EO	yes	25	96
11	♂	70	oropharynx	pT3 pN1 L1 V0 Pn0 R0	G3	II	SCC	pos.	EO	yes	10	80
12	♂	63	oropharynx	pT2 pN3b L1 V1 Pn1 R0	G2	IVB	SCC	neg.	EO	yes	30	97
13	♂	73	paranasal sinuses	pT4a pN0 L0 V0 Pn1 R1	G2	IVA	ACC	/	MFR	yes	15	161
14	♂	57	oral cavity	pT1 pN0 L1 V0 Pn0 R0	G2	I	SCC	neg.	EO	yes	35	161
15	♂	38	oropharynx	pT4a pN3b L0 V0 Pn0 R1	G2	IVB	SCC	neg.	EO, LP	yes	30	119

Table S1. Patient characteristics. Specifying the patient’s carcinoma (SCC – squamous cell carcinoma or ACC – adenoid cystic carcinoma) and tumor resection method (MS – mandibular splitting, EO – enoral, LP – lateral pharyngotomy, or MFR – midfacial resection).

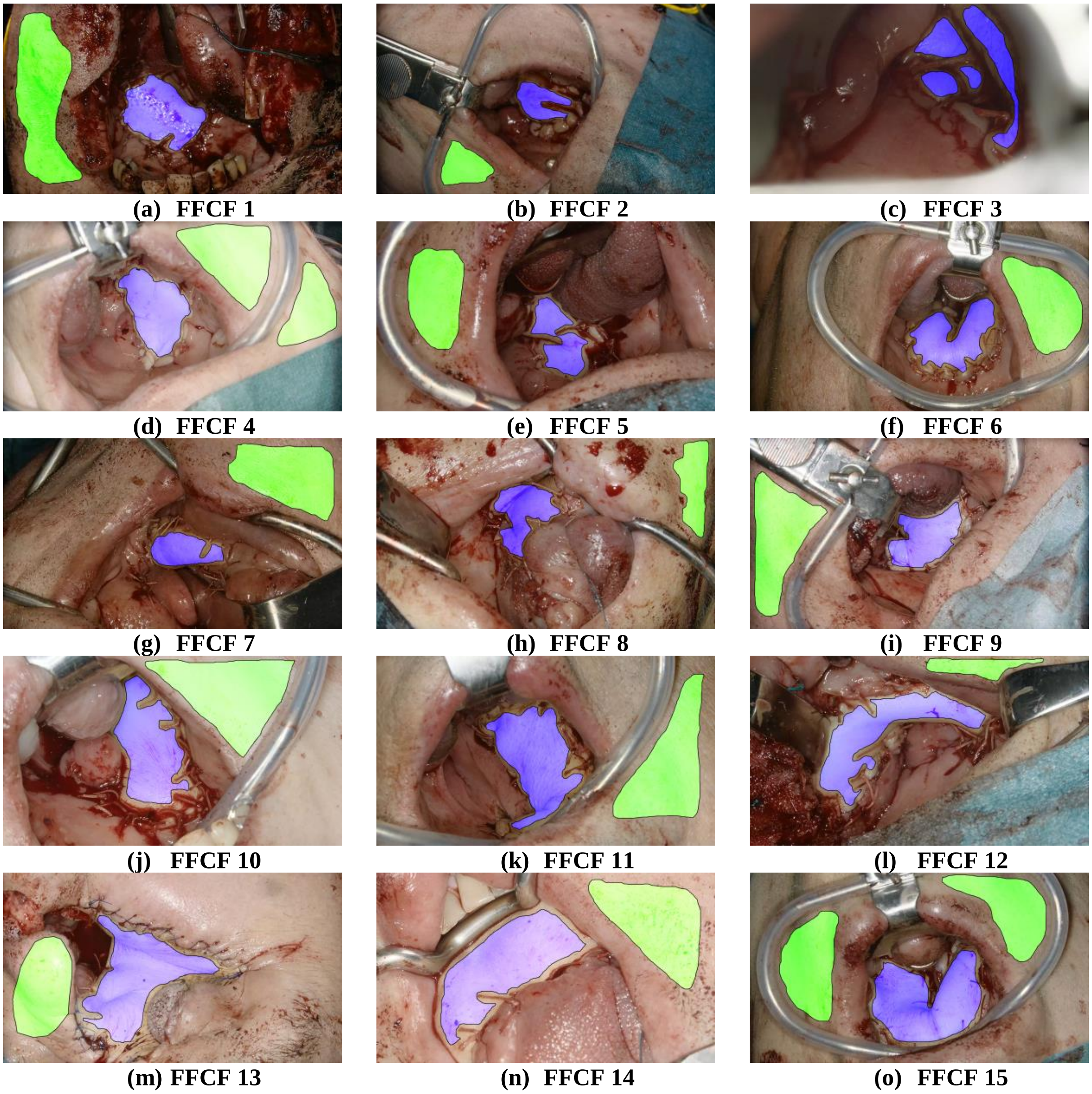

Fig. S1. Annotations of analyzed flap region-of-interest (blue) and skin reference region (green) for all cases (of third recording flap perfusion). For (c) FFCF 3, it was not possible to define a continuously well perfused reference region.

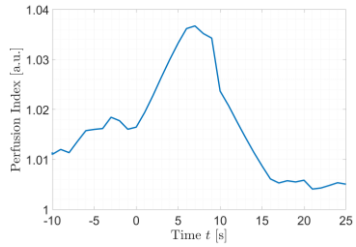

(a) FFCF 1

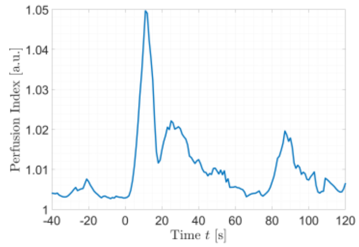

(b) FFCF 2

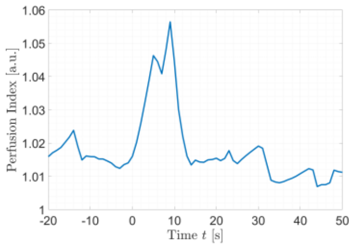

(c) FFCF 3

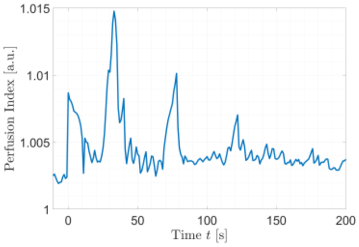

(d) FFCF 4

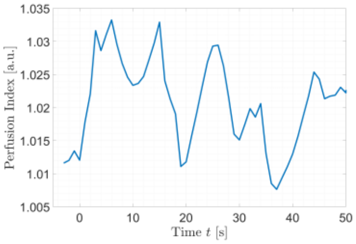

(e) FFCF 5

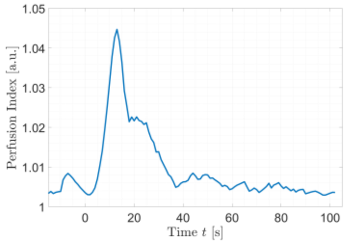

(f) FFCF 6

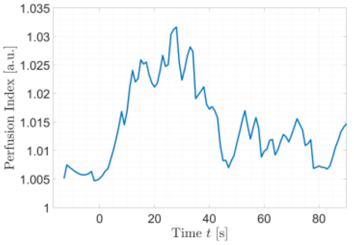

(g) FFCF 7

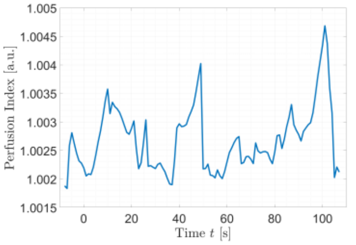

(h) FFCF 8

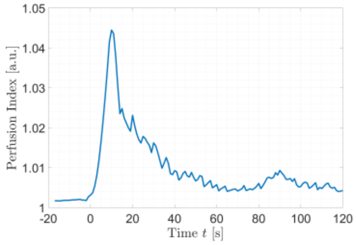

(i) FFCF 9

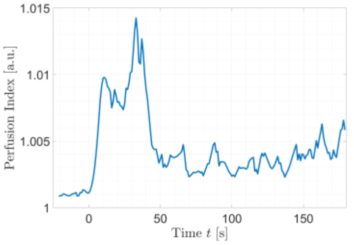

(j) FFCF 10

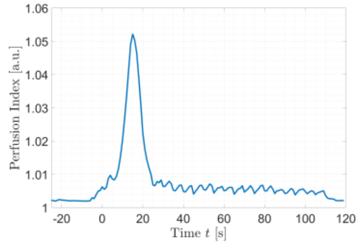

(k) FFCF 11

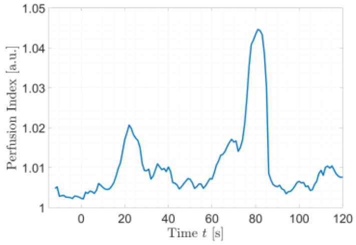

(l) FFCF 12

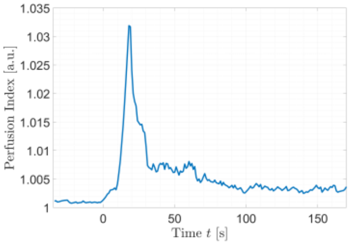

(m) FFCF 13

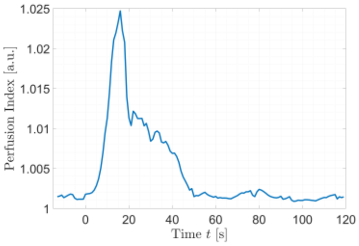

(n) FFCF 14

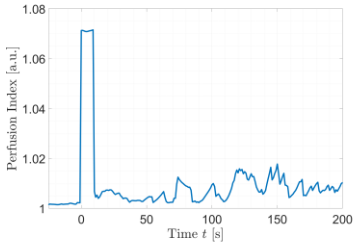

(o) FFCF 15

Fig. S2 Perfusion Index (PI) for all cases (of third recording flap perfusion). In (o) FFCF 15, illumination changes right at the time of reperfusion affect PI. These changes result in an amplitude of the signal adding onto the normal reperfusion for the time of the illumination changes.

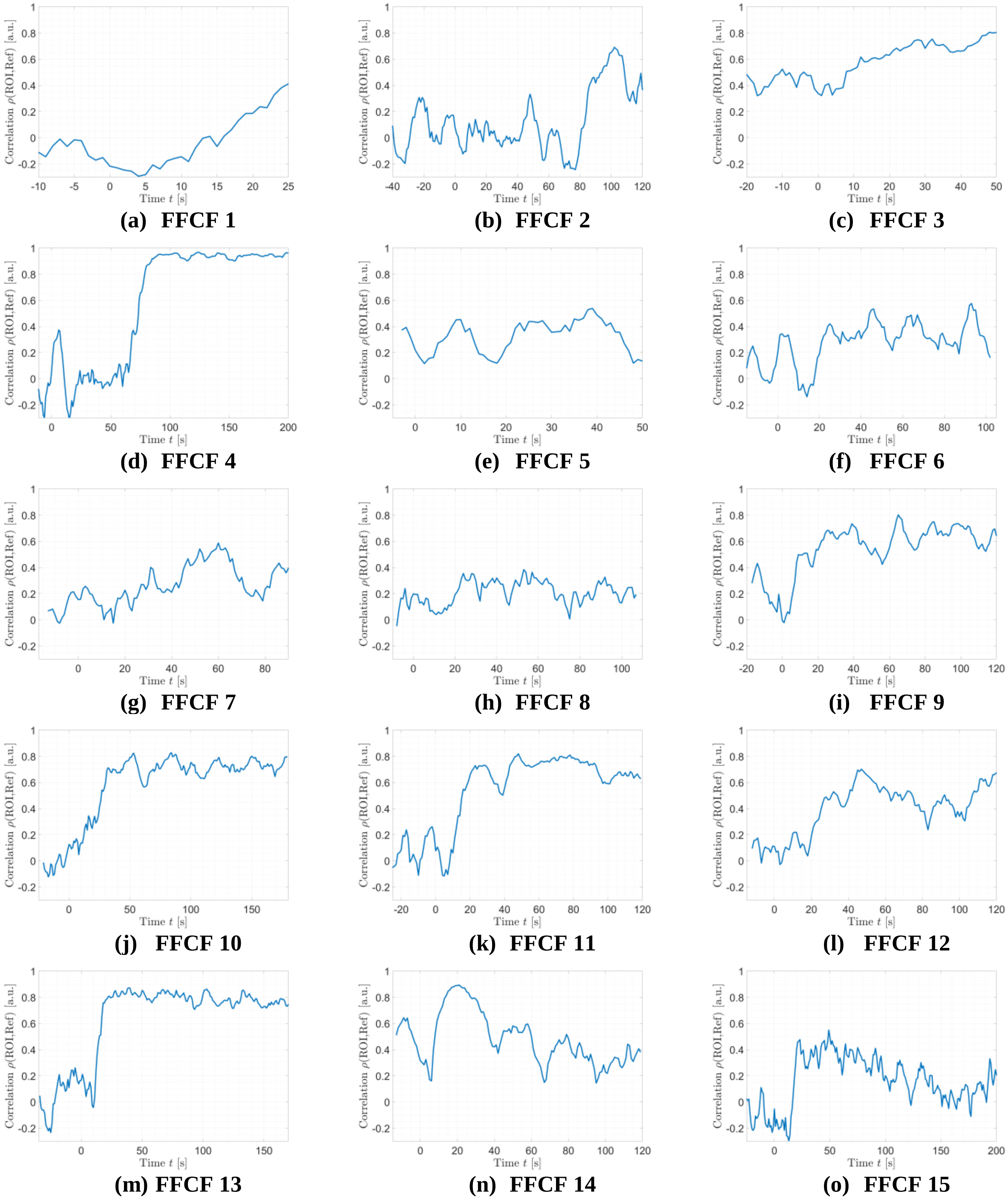

Fig. S3. Correlation ρ between region-of-interest and reference data for all cases (of third recording flap perfusion).

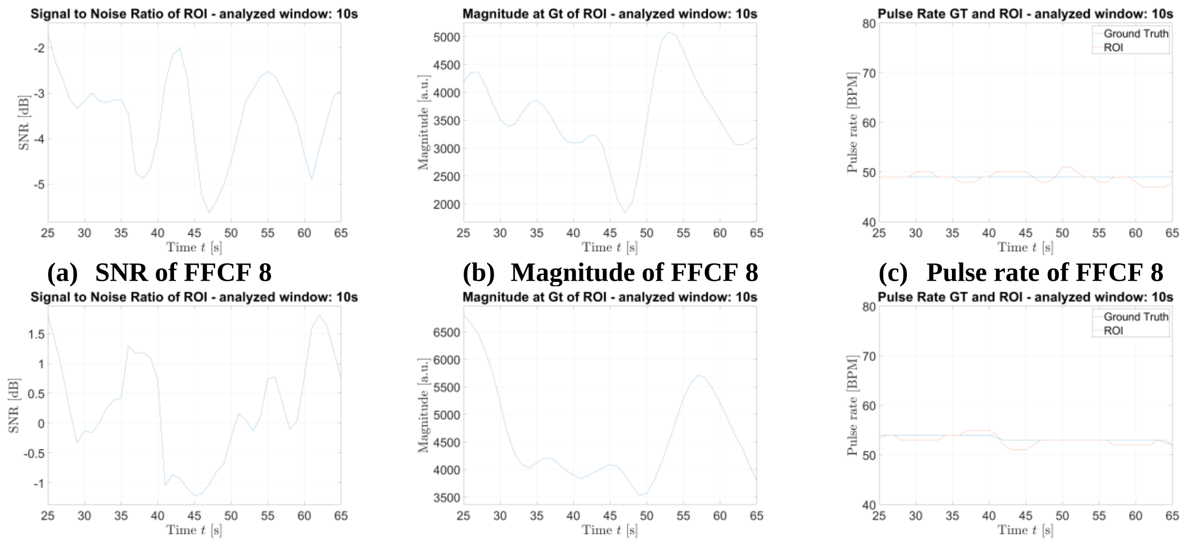

(a) SNR of FFCF 8 **(b) Magnitude of FFCF 8** **(c) Pulse rate of FFCF 8**

(d) SNR of FFCF 14 **(e) Magnitude of FFCF 14** **(f) Pulse rate of FFCF 14**

Fig. S4. Post-surgical (one hour after reperfusion) analysis of SNR and magnitude as well as pulse rate extraction for the failed flap (FFCF 8) and a flap showing long time of vasospasms during surveillance (FFCF 14). The ground truth data in (c) and (f) are taken from the patient monitor connected to the patient.

Movie S1. Visualization of local fasciocutaneous flaps behavior. The visualization of the continuous perfusion behavior is shown of FFCF 13. At the beginning, perfusion index (PI) and rPPG correlation ρ are low (colored from blue to greenish). At the time of reperfusion, PI shows its characteristic peak (in red) and at the same time, the correlation ρ starts to increase (becoming red over the entire flap) showing continuous perfusion. This visualization corresponds to the plots in Figs. S2 (m) and S3 (m).
